# Traumatic Brain Injury Exposure Lowers Age of Cognitive Decline in AD and Non-AD Conditions

**DOI:** 10.3389/fneur.2021.573401

**Published:** 2021-05-12

**Authors:** Diego Iacono, Sorana Raiciulescu, Cara Olsen, Daniel P. Perl

**Affiliations:** ^1^Department of Defense/Uniformed Services University (DoD/USU) Brain Tissue Repository & Neuropathology Program, Uniformed Services University of the Health Science (USU), Bethesda, MD, United States; ^2^Department of Neurology, F. Edward Hébert School of Medicine, Uniformed Services University of the Health Science (USU), Bethesda, MD, United States; ^3^Department of Pathology, F. Edward Hébert School of Medicine, Uniformed Services University of the Health Science (USU), Bethesda, MD, United States; ^4^Neuroscience Graduate Program, Department of Anatomy, Physiology, and Genetics, F. Edward Hébert School of Medicine, Uniformed Services University of the Health Science (USU), Bethesda, MD, United States; ^5^The Henry M. Jackson Foundation for the Advancement of Military Medicine (HJF), Bethesda, MD, United States; ^6^Complex Neurodegenerative Disorders, Neurodegenerative Disorders Clinic, National Institute of Neurological Disorders and Stroke (NINDS), NIH, Bethesda, MD, United States; ^7^Department of Preventive Medicine and Biostatistics, F. Edward Hébert School of Medicine, Uniformed Services University of the Health Science (USU), Bethesda, MD, United States

**Keywords:** TBI, earlier-onset, neurodegenerative disorders, APOE genotype, cognitive decline

## Abstract

We aimed to detect the possible accelerating role of previous traumatic brain injury (TBI) exposures on the onset of later cognitive decline assessed across different brain diseases. We analyzed data from the National Alzheimer's Coordinating Center (NACC), which provide information on history of TBI and longitudinal data on cognitive and non-cognitive domains for each available subject. At the time of this investigation, a total of 609 NACC subjects resulted to have a documented history of TBI. We compared subjects with and without a history of previous TBI (of any type) at the time of their first cognitive decline assessment, and termed them, respectively, TBI+ and TBI– subjects. Three hundred and sixty-one TBI+ subjects (229 male/132 female) and 248 TBI– subjects (156 male/92 female) were available. The analyses included TBI+ and TBI– subjects with a clinical diagnosis of Mild Cognitive Impairment, Alzheimer's disease, Dementia with Lewy bodies, Progressive supranuclear palsy, Corticobasal degeneration, Frontotemporal dementia, Vascular dementia, non-AD Impairment, and Parkinson's disease. The data showed that the mean age of TBI+ subjects was lower than TBI– subjects at the time of their first cognitive decline assessment (71.6 ± 11.2 vs. 74.8 ± 9.5 year; *p* < 0.001). Moreover, the earlier onset of cognitive decline in TBI+ vs. TBI– subjects was independent of sex, race, attained education, APOE genotype, and importantly, clinical diagnoses. As for specific cognitive aspects, MMSE, Trail Making Test part B and WAIS-R scores did not differ between TBI+ and TBI– subjects, whereas Trail Making Test part A (*p* = 0.013) and Boston Naming test (*p* = 0.008) did. In addition, data showed that neuropsychiatric symptoms [based on Neuropsychiatry Inventory (NPI)] were much more frequent in TBI+ vs. TBI– subjects, including AD and non-AD neurodegenerative conditions such as PD. These cross-sectional analyses outcomes from longitudinally-assessed cohorts of TBI+ subjects that is, subjects with TBI exposure before the onset of cognitive decline in the contest of different neurodegenerative disorders and associated pathogenetic mechanisms, are novel, and indicate that a previous TBI exposure may act as a significant “*age-lowering*” factor on the onset of cognitive decline in either AD and non-AD conditions independently of demographic factors, education, APOE genotype, and current or upcoming clinical conditions.

## Introduction

During the last few decades, there has been a flourishing of investigations focusing on the possible long-term neurological and psychiatric sequelae generated by traumatic brain injury (TBI) events (1–6). Researchers have sought possible direct pathogenetic links between single-TBI (sTBI) or repetitive-TBI (rTBI) and neurodegenerative disorders culminating, for example, in conditions like Alzheimer's disease (AD) (7–9), Parkinson's disease (PD) (10, 11), Frontotemporal Dementia/Amyotrophic Lateral Sclerosis (FTD/ALS) (12, 13), Dementia with Lewy Bodies (DLB) (14), and other (15). While clinico-epidemiological evidences for direct links between rTBI and neurodegeneration have been known for decades (dementia pugilistica and pugilistic parkinsonism, for example) (16–18), only recently, a more systematic investigative approach on long-term TBI consequences have been implemented, especially in terms of search for meaningful clinico-neuropathological correlations between TBI and specific neurodegenerative mechanisms (4, 19, 20). In addition, a renewed attention for medically-relevant links between TBI and neurodegeneration has been boosted by mass media news reporting an increased frequency of different neurological and psychiatric manifestations (21, 22) and early deaths, including suicides, among professional American Football players *(http://time.com/4871597/degenerative-brain-disease-cte-football)* (23). In particular, these contact-sports related cases have been associated with Chronic Traumatic Encephalopathy (CTE) (24), a peculiar type of brain pathology recognized many decades ago already (16). However, an even more dramatic event has been associated with TBI exposure that is, an increased number of military service members manifesting severe neurological and psychiatric disorders after combat-related TBI exposure occurred during periods of deployment in war zones in the Middle-East [Operation Enduring Freedom (OEF), Operation Iraqi Freedom (OIF), and Operation New Dawn (OND)] (25–29). These social and historical factors have recently stimulated researchers to investigate, more accurately, on the possible links between TBI and neurodegeneration. As a result, an increasingly improved understanding of the possible interconnected pathogenetic mechanisms between TBI and neurodegenerative consequences has been lately produced (30).

In this study, we aimed to observe if: (a) a previous history of sTBI or rTBI, of any type and number, with or without loss of consciousness (LOC), could accelerate the manifestation of cognitive decline as assessed across different clinical diagnosis: Mild Cognitive Impairment (MCI), Dementia of different types (Dementia), and non-AD cognitive impairment (Impaired); (b) demographic, educational or APOE genotype could differ between TBI+ and TBI– subjects and across all three conditions (MCI, Dementia, Impaired); (c) time intervals between the first cognitive decline assessment and death could differ between TBI+ and TBI– subjects across all clinical condition (MCI, Dementia, Impaired) and be possibly correlated with a higher frequency of a specific APOE genotype or allele.

## Materials and Methods

To achieve our aims, we interrogated one of the largest datasets on aging and dementia database currently available—the National Alzheimer's Coordinating Center (NACC) Uniform Data Set (UDS) (https://www.alz.washington.edu). NACC-UDS's data collection is an active clinic-based population database gathering information on patients enrolled across different AD centers participating in the NACC consortium. The obtained data were all non-identifiable medical encounter data, which included prospectively collected information on neurological and psychiatric diagnoses, other medical conditions, demographics, attained educational levels, and genetic information. These NACC-UDS's data represent a set of information particularly useful for identifying possible general clinico-epidemiological aspects of subjects exposed to different types of TBI. Moreover, these data are extremely valuable to analyze cohorts of subjects exposed to TBI that later manifested neurological and psychiatric diseases, in particular cognitive decline, in the context of different subjacent neurodegenerative processes such as AD, DLB, or PD (4).

Briefly, the NACC database was established by the National Institute on Aging (NIA) in 1999 to facilitate collaborative research on various aspects of dementia. Using data collected from the NIA-funded Alzheimer's Disease Centers (ADCs) across the United States NACC has developed and maintains a large database of standardized clinical and neuropathological research data. NACC data used for research are IRB-approved from ethical standards committees to conduct this type of study.

For the above-described aims of this investigation, a specific set of data was created by interrogating the NACC database to gather information on subjects that reported a history of TBI together with data on physical, neurological, motor, cognitive, and behavioral evaluation at the moment of their first cognitive decline assessment. These TBI subjects could have been enrolled in any AD center participating in the NACC.

The inclusion selection criteria for this study were:

1) 18+ male or female subject of any race with a history of one or more TBI, with or without LOC, and cognitive decline assessed at any visit during their period of enrollment in any of the participating AD Research Center of the NACC Consortium;
2) Availability of TBI time points (including time points about TBI before or after any cognitive, neurological, or psychiatric deficit);
3) Availability of demographic, education, APOE genotype, cognitive, neurological, and psychiatric data;

The exclusion selection criteria for this study were:

1) Less than 18 years of age;
2) No availability of reported TBI history or time points;
3) Mendelian mutations or chromosomal deletions;

As for TBI related NACC variables the following variables were preliminary considered: TBI, TBIBRIEF, TRAUMBRF, TBIEXTEN, TRAUMEXT, TBIWOLOS, TRAUMCHR, TBIYEAR. The full set of variables considered in our analyses is reported in Supplementary Data 1. Each of these variables is fully described in the NACC dictionary (https://www.alz.washington.edu/WEB/forms_uds.html). Preliminarily, we harmonized all available TBI related data to obtain uniform information about presence or absence of TBI across all available NACC data versions (V1–V3) at the time of the study. In particular, we have used the variable BRNINJ for version V1, V2, V3, which was the only variable covering TBI events across all three versions and included the variable TBI, which was used in V3 only. The NACC dataset was not ideated to specifically collect TBI data, and information about clinical severity of TBI or exact age of TBI occurrence, are mostly lacking. Despite that, and to satisfy our primary aims, we decided to mainly focus on the presence of TBI before or after the first formally assessed cognitive decline as accurately documented in the NACC dataset.

Based on the above-described inclusion/exclusion selection criteria, by May 2019, a total of 609 (11.4%) out of 5,336 NACC subjects with a reported history of TBI were available. Three hundred and sixty-one subjects (229 male/132 female) had a history of a previous TBI exposure, single or repetitive, with or without LOC, at the time of their first cognitive decline assessment (TBI+ subjects); and 248 subjects (156 male/92 female) had no history of a previous TBI exposure at the time of their first cognitive decline assessment (TBI– subjects). The sizes of these two TBI cohorts (TBI+ and TBI–cohorts) are results obtained from standardized questions about the history of TBI for each enrolled subject across different longitudinal studies. During the initial and following physical examination/health data collection each participant was asked if he/she had ever experienced a head trauma, including concussions or sub-concussions events, if there was loss of consciousness (LOC) or not, and if present, estimated LOC duration. Table 1 summarizes basic demographic, sex, race, and attained educational levels data of both TBI+ and TBI– subjects NACC cohorts available for this investigation.

**Table 1:** Demographic data at the time of the first cognitive decline assessment.

	**TBI– (*n =* 248)**	**TBI+ (*n =* 361)**	***p*-value for row main effect from two-way ANOVA**
**Age**	74.87 (9.58)	71.62 (11.24)	<0.001^*^
**Sex**			0.96
*Male TBI– (n = 156); TBI+ (n = 229)*	74.14 (9.41)	70.83 (11.5)	
*Female TBI– (n = 92); TBI+ (n = 132)*	76.11 (9.79)	72.99 (10.6)	
**Race**			^**^0.056
*African American TBI– (n = 32); TBI+ (n = 4)*	76.96 (6.60)	70.09 (11)	
*American Indian TBI– (n = 1); TBI+ (n = 4)*	63 (–)	50 (14.4)	
*Asian TBI– (n = 8); TBI+ (n = 3)*	78.5 (10.3)	73.3 (18.5)	
*Multiracial TBI– (n = 7); TBI+ (n = 21)*	71.83 (10.5)	66.94 (14.4)	
*White TBI– (n = 199); TBI+ (n = 289)*	74.53 (9.89)	72.48 (10.7)	
**Education**			0.45
*High school or less TBI– (n = 62); TBI+ (n = 103)*	76.31 (10.1)	71.23 (11.6)	
*Bachelor's Degree TBI– (n = 108); TBI+ (n = 140)*	74.51 (9.07)	71.91 (12.1)	
*Master's Degree TBI– (n = 50); TBI+ (n = 82)*	74.32 (9.26)	70.68 (9.34)	
*Doctorate Degree TBI– (n = 28); TBI+ (n = 33)*	74.07 (10.9)	74.09 (11.1)	

* *p-value represents t-test between TBI– and TBI+*.

** *Due to the paucity of the American Indian subjects in this cohort, we included them in Multiracial category group*.

*Interactions between sex, race, education levels in all two-way ANOVAs were not significant*.

*The table shows the main demographic data found in TBI+ (subjects with a previous history of TBI at the time of their first cognitive decline assessment) and TBI– (subjects without a previous history of TBI at the time of their first cognitive decline assessment)*.

At the time of the first cognitive decline assessment for both TBI+ and TBI– subjects, the following battery of cognitive tests were conducted: Mini Mental State Examination (MMSE) (31), Trail Making Test Part A and Part B (32), Boston Naming Test (33), and WAIS-R digit (34). A clinical diagnosis of MCI (including amnesic and non-amnesic MCI), Dementia (including AD, DLB, FTD, VaD, PSP, CBD, TBI, dementia associated with systemic disease) and Impaired (non-AD Impairment) were, respectively, defined by the following NACC's variables: NACCUDSD (NACC-Uniform Data Set Dictionary) = 3 (MCI), = 4 (Dementia), and = 2 (Impaired). As per NACC's definition, the diagnosis for Impaired subjects (NACCUDSD = 2) was applied when “Subjects who are cognitively impaired but who do not meet the criteria for MCI.” To assess neuropsychiatric manifestations, the Neuropsychiatric Inventory (NPI) was employed (35).

The clinical diagnostic criteria used for each condition considered in this study [MCI, specific type of Dementia, and non-AD Impairment (Impaired)] followed the clinical criteria employed by NACC as described in the NACC glossary (https://www.alz.washington.edu).

Furthermore, based on a recent investigation showing a possible direct link between TBI with LOC and autopsy-verified Lewy body pathology accumulation, progression of parkinsonism, and Parkinson's disease (PD) (36), in addition to the MCI, Dementia, and Impaired status, we sought differences between TBI+ vs. TBI– subjects with a diagnosis of PD across all examined conditions (MCI, Dementia, Impaired). In addition, TBI+/PD and TBI–/PD subjects' mean score on the UPDRS-motor assessment (UPDRS-Part III) at the time of their first cognitive decline assessment were also analyzed. In the PD subjects cohorts, in order to minimize potential confounding factors due to other overlapping pathologies different from the Lewy body pathology such as AD pathology (MCI/TBI+/PD or AD-Dementia/TBI+/PD), we focused principally on the possible differences between TBI+/PD/Impaired vs. TBI–/PD/Impaired subjects.

### Data Availability

Anonymized data will be shared by request from qualified investigators.

### Statistics

Differences between specific demographic variables and TBI status in the age of onset at time of the first assessment, UPDRS-motor scores, and NPI Severity scores were elaborated using two-way analysis of variance (two-way ANOVA). Variation in mean cognitive test scores between the two TBI groups was analyzed using *t*-tests, with adjustments for multiple comparisons using Tukey's method, where appropriate. Further analyses of cognitive test scores for the three clinical conditions (MCI, Dementia, Impaired) in the TBI+ group were analyzed *via* one-way analysis of variance (one-way ANOVA) followed by corrected pairwise comparisons. Differences among TBI groups in terms of mean time interval from the first declined cognitive assessment to death were also calculated using *t*-tests. Genotype and single allelic frequencies by TBI status within each clinical condition were explored using a test of proportions. APOE frequencies were further compared within the TBI+ group across the condition groups using a pairwise comparison of proportions, adjusting for multiple comparisons using the Holm method. All analyses were conducted in the statistical software R (37).

## Results

The statistical analyses showed that at the time of the first cognitive decline assessment TBI+ subjects were significantly younger in comparison to TBI– subjects (*p* < 0.001). On average, TBI+ subjects were 3.25 years younger than TBI– subjects across all clinical conditions (MCI, Dementia, and Impaired). Moreover, the *TBI age-lowering effect* was observed across all considered clinical conditions in both sexes, across all races, and any educational level attained. In addition, no meaningful interactions between sexes or among races and educational levels were found between TBI+ vs. TBI– subjects. Table 1 summarizes all main demographic data and resulting *p*-values found in TBI+ and TBI– group.

**Figure 1:**
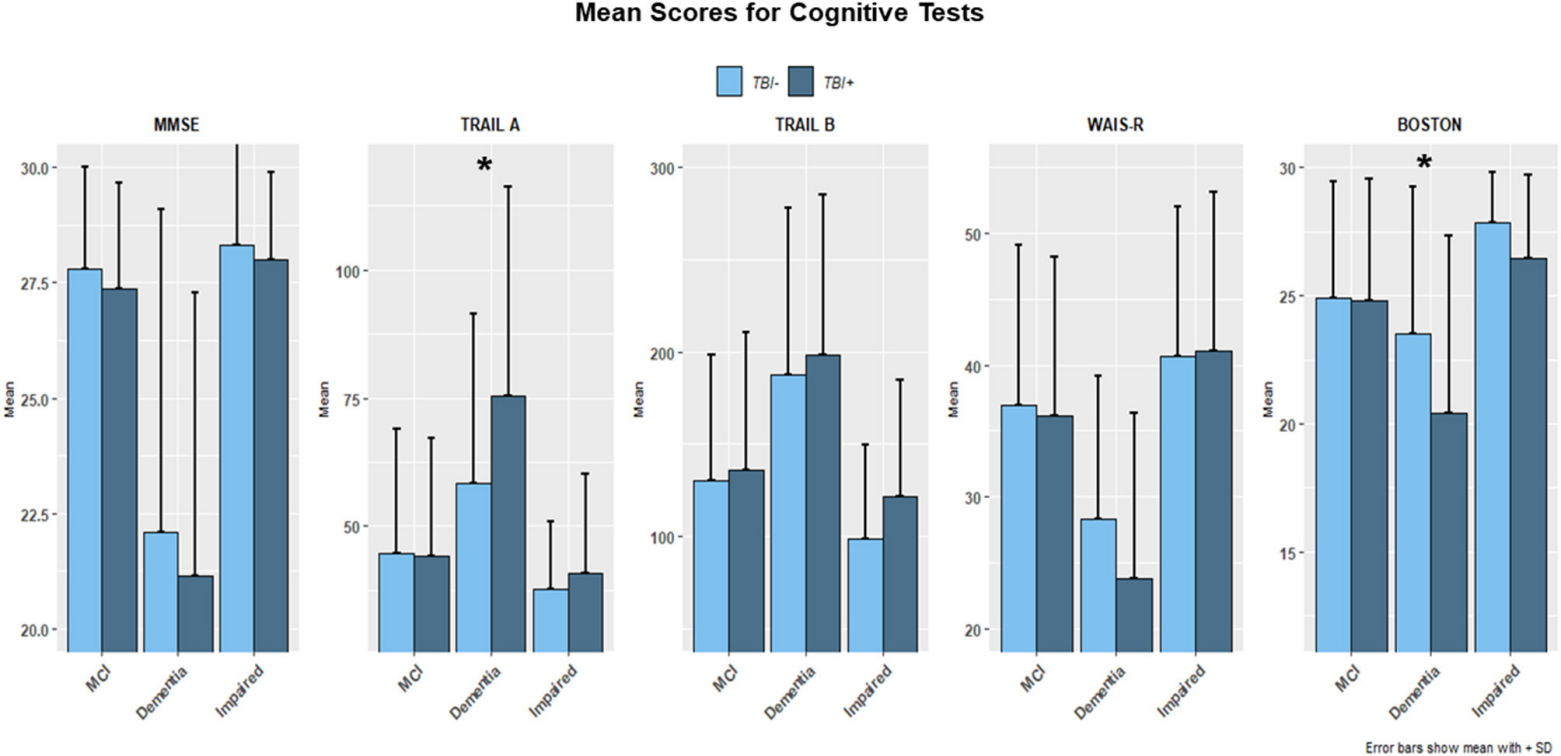
The figure shows bar plots of the mean scores for the Mini Mental Status Examination (MMSE), Trail Making Test part A and B (TMT-A, TMT-B), WAIS-R scale and Boston Naming Test for both TBI+ (subjects with a previous history of TBI at the time of their first cognitive decline assessment) and TBI– (subjects without a previous history of TBI at the time of their first cognitive decline assessment) as clustered by their associated clinical diagnosis at the time of their first cognitive decline assessment. MCI, Mild cognitive impairment, Dementia [including Alzheimer's disease (AD), Dementia with Lewy bodies (DLB), Progressive supranuclear palsy (PSP), Corticobasal degeneration (CBD), Frontotemporal Dementia (FTD), Vascular dementia (VaD), Traumatic brain Injury (TBI), normal pressure hydrocephalus (NPH), Depression, Cognitive decline due to systematic disease or medical illness], non-AD Impairment (Impaired). For TMT-A (*p* = 0.013) and Boston Naming test (*p* = 0.008) a significance was found TBI+ vs. TBI– groups. *indicates the presence of statistical significance (*p*-values <0.05).

As for specific cognitive test scores at the time of the first cognitive decline assessment, no differences between TBI+ and TBI– subjects were found, except for the Trail Making Test Part A and Boston Naming Test scores, which, respectively, showed longer times spent for completion of the text (TMT Part A; *p* = 0.013) and lower naming scores (*p* = 0.008) in the TBI+ vs. TBI– group. Furthermore, when considering possible differences across TBI+ subjects only and all clinical conditions (TBI+/MCI, TBI+/Dementia, TBI+/Impaired), differences for cognitive tests were detected between TBI+/Impaired and TBI+/Dementia (*p* < 0.001), TBI+/MCI and TBI+/Dementia (*p* < 0.001), but not between TBI+/Impaired and TBI+/MCI (*p* = 0.65), with the only exception represented by WAIS-R Digit Symbol test scores (*p* = 0.046). Figure 1 shows bar plots for each single type of cognitive test analyzed, mean scores, and *p*-values for all comparisons between TBI+ vs. TBI– group.

**Figure 2:**
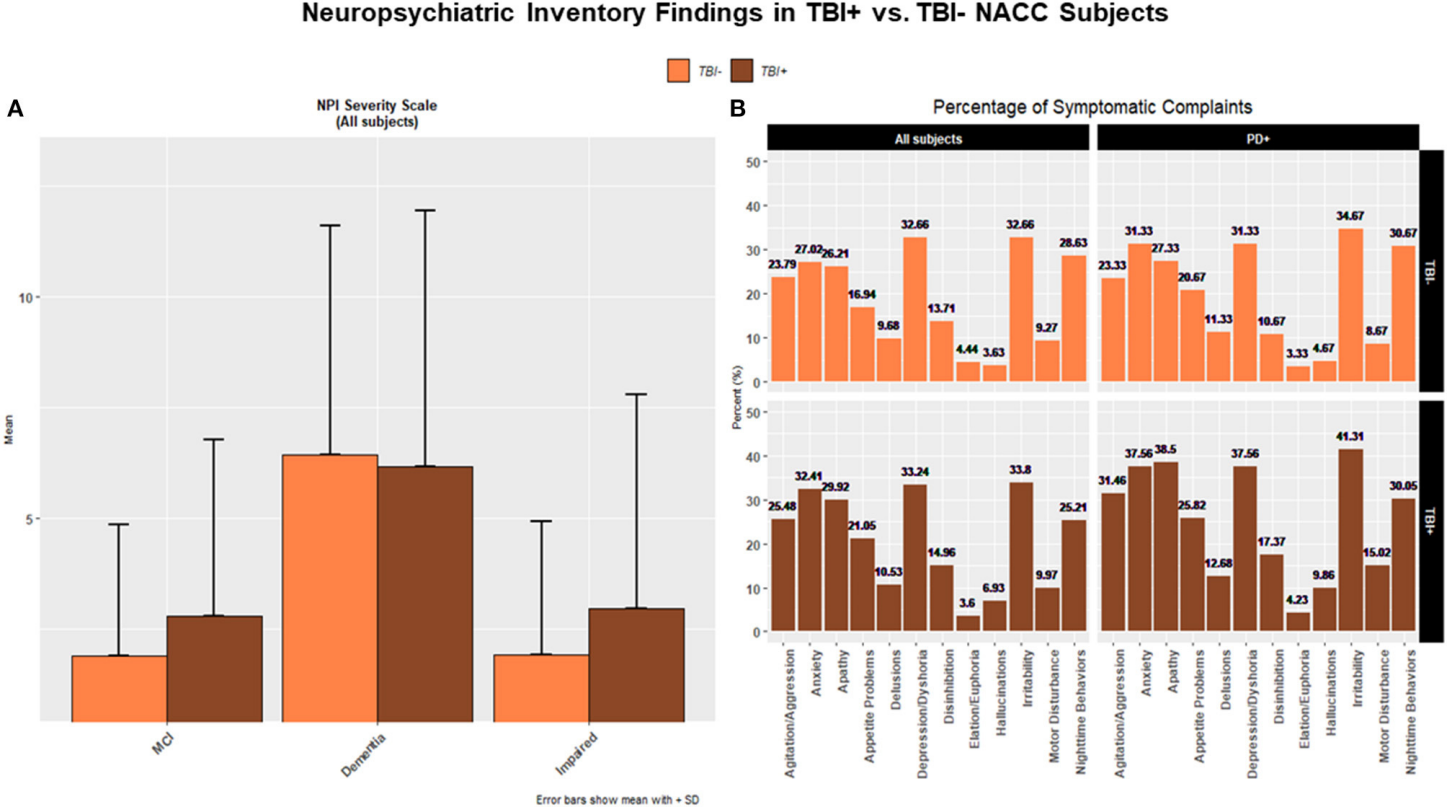
**(A)** The figure shows bar plots of the mean scores for the Neuropsychiatric Inventory (NPI) for both TBI+ (subjects with a previous history of TBI at the time of their first cognitive decline assessment) and TBI– (subjects without a previous history of TBI at the time of their first cognitive decline assessment) as clustered by their associated clinical diagnosis at the time of their first cognitive decline assessment. MCI, Mild cognitive impairment, Dementia [including Alzheimer's disease (AD), Dementia with Lewy bodies (DLB), Progressive supranuclear palsy (PSP), Corticobasal degeneration (CBD), Frontotemporal Dementia (FTD), Vascular dementia (VaD), Traumatic brain Injury (TBI), normal pressure hydrocephalus (NPH), Depression, Cognitive decline due to systematic disease or medical illness], non-AD Impairment (Impaired). A trend for TBI+ subjects with a diagnosis of MCI and Impaired to have a higher frequency of neuropsychiatric disorders and symptoms as mostly listed in the NPI was observed. **(B)** The histograms show percentages (%) of frequency for NPI-based neuropsychiatric disorders in TBI– and TBI+ groups across all subjects (upper and lower left panels) and PD subjects (upper and lower right panels).

As for the NPI scores, a higher frequency of psychiatric manifestations through all conditions, and for almost all NPI items, was found in TBI+ vs. TBI– subjects (Figure 2). Moreover, TBI+ vs. TBI– subjects were more often diagnosed with neuropsychiatric symptoms across MCI, Dementia, and Impaired status. Likewise, TBI+/PD vs. TBI–/PD subjects were more often affected by almost all neuropsychiatric symptoms as itemized in the NPI. In this case, however, the statistical comparison did not reach significance probably due to the small cohorts' size. In general, though, the trend for TBI+ subjects to manifest more frequently neuropsychiatric symptoms in comparison to TBI– subjects was a constant observation across all clinical diagnoses (MCI, Dementia, Impaired).

**Figure 3:**
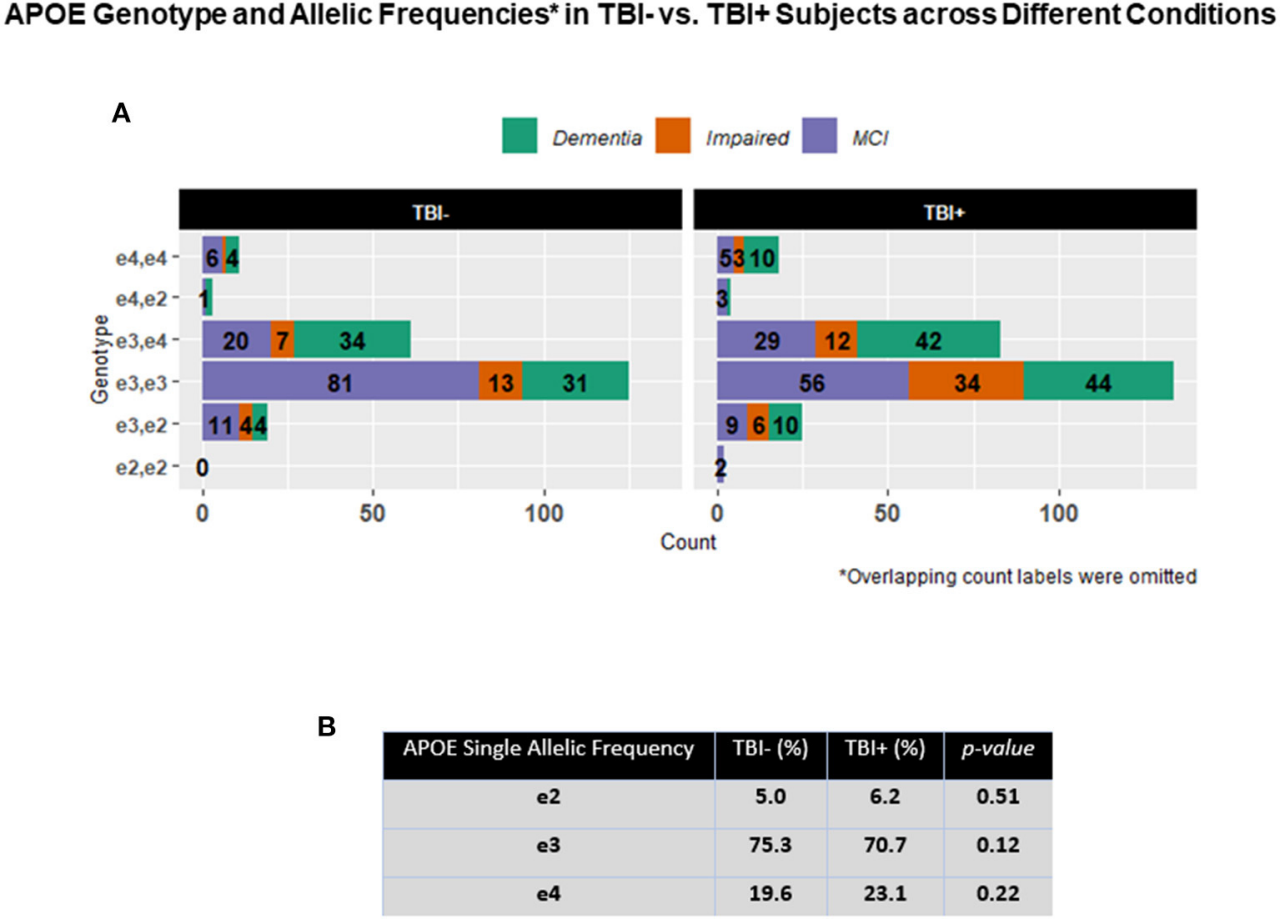
**(A)** The figure shows horizontal stacked bars for the description of the relative frequencies of the different APOE in both TBI+ (subjects with a previous history of TBI at the time of their first cognitive decline assessment) and TBI– (subjects without a previous history of TBI at the time of their first cognitive decline assessment) as clustered by their associated clinical diagnosis at the time of their first cognitive decline assessment. MCI: Mild cognitive impairment, Dementia [including Alzheimer's disease (AD), Dementia with Lewy bodies (DLB), Progressive supranuclear palsy (PSP), Corticobasal degeneration (CBD), Frontotemporal Dementia (FTD), Vascular dementia (VaD), Traumatic brain Injury (TBI), normal pressure hydrocephalus (NPH), Depression, Cognitive decline due to systematic disease or medical illness], non-AD Impairment (Impaired). **(B)** The table in the figure shows the general APOE allelic frequencies (APOE2, E3, E4) in both TBI+ and TBI– subjects in the analyzed NACC's TBI cohort.

APOE genotype and single APOE allelic frequencies across TBI+ and TBI– groups did not show differences in comparison to APOE genotype and single APOE allelic frequencies as measured in the general Caucasian population. These APOE findings confirmed then that also in these NACC TBI subjects cohorts (the majority of which were Caucasians), the E3 is the most frequent allele followed by the allele E4 and E2 of the APOE gene (38, 39). Nonetheless, when considering pairwise proportion tests with multiple comparisons adjustment for the E4 allelic frequency through the entire TBI+ subjects group only, a difference was detected in TBI+/Impaired vs. TBI+/Dementia group (*p* = 0.044), but not between TBI+/Impaired and TBI+/MCI, or between TBI+/MCI and TBI+/Dementia (Figure 3).

Interestingly, the mean interval of time between the first cognitive decline assessment and death was shorter in TBI+ vs. TBI– subjects across all clinical conditions (*p* < 0.001), in TBI+ vs. TBI– subjects with MCI (*p* = 0.003), and in TBI+ vs. TBI– with Dementia (*p* < 0.001). Importantly, a shortened mean interval of time between the first cognitive decline assessment and death in TBI+ vs. TBI– subjects was not associated with a higher frequency of any APOE genotype or single APOE allelic frequency across all conditions. Table 2 summarizes Cognitive Decline-Death time intervals and single APOE allelic distributions in TBI+ and TBI– across all clinical conditions cumulatively considered (MCI+Dementia+Impaired) and across each condition (MCI, Dementia, Impaired).

**Table 2:** Cognitive decline-death intervals and allelic APOE frequencies in TBI+ vs. TBI– subjects.

	**All Conditions (MCI+AD+Impaired)**		**MCI**		**Dementia**		**Impaired**	
	**TBI–**	**TBI+**	**TBI–**	**TBI+**	**TBI–**	**TBI+**	**TBI–**	**TBI+**
Mean cognitive decline-death interval (years)	5.94	3.63^*^	6.26	4.40^*^	5.58	3.36^*^	6.38	4.33
APOE2 allelic frequency (%)	5.0	6.2	5.0	7.6	4.0	5.1	8	5.4
APOE3 allelic frequency (%)	75.3	70.7	81.0	72.1	66.6	65.4	74	78.1
APOE4 allelic frequency (%)	19.6	23.1	13.8	20.1	29.3	29.4	16	16.3

*The table shows the mean intervals of time between the first cognitive decline assessment and death for all TBI+ and TBI– subjects across all cognitive conditions (MCI, AD, Impaired) at that timepoint as either cumulatively (MCI+AD+Impaired) or separately (MCI, AD, Impaired) considered*.

* *Indicates the presence of statistical significance (p < 0.05) for the mean intervals of time (in years) between the first cognitive decline assessment and death for MCI and AD subjects with (TBI+) and without (TBI–) history of traumatic brain injury (TBI) before that first cognitive assessment*.

**Figure 4:**
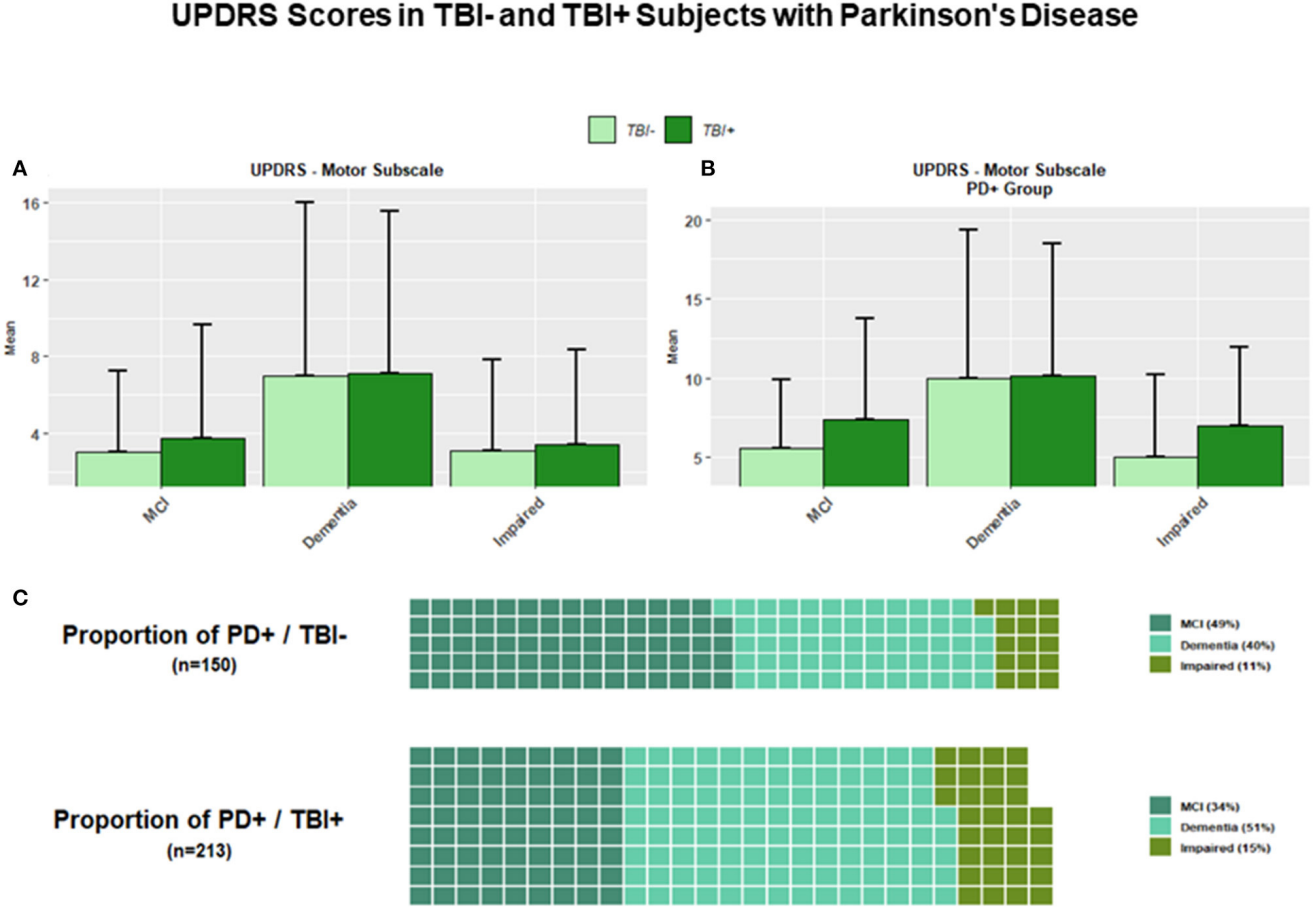
**(A)** The figure shows bar plots for the UPDRS-Motor scale across all TBI+ (subjects with a previous history of TBI at the time of their first cognitive decline assessment) and TBI– (subjects without a previous history of TBI at the time of their first cognitive decline assessment) as clustered by their associated clinical diagnosis at the time of their first cognitive decline assessment. MCI, Mild cognitive impairment, Dementia [including Alzheimer's disease (AD), Dementia with Lewy bodies (DLB), Progressive supranuclear palsy (PSP), Corticobasal degeneration (CBD), Frontotemporal Dementia (FTD), Vascular dementia (VaD), Traumatic brain Injury (TBI), normal pressure hydrocephalus (NPH), Depression, Cognitive decline due to systematic disease or medical illness], non-AD Impairment (Impaired). **(B)** The figure shows bar plots for the UPDRS-Motor scale in TBI+ and TBI– subjects with a diagnosis of Parkinson's disease (PD) at the time of their first cognitive decline assessment. Both TBI+/PD/MCI and TBI+/PD/Impaired subjects showed higher mean scores vs. TBI–/PD/MCI and TBI–/PD/Impaired subjects. **(C)** The two waffle graphs show the counts and corresponding proportions of MCI, Dementia and Impaired subjects with a diagnosis of PD with (TBI+) and without (TBI–) a history of TBI prior to their first cognitive decline assessment.

As for the TBI+/PD and TBI–/PD subgroups, the results showed that at the time of the first cognitive impairment TBI+/PD subjects were significantly more numerous than TBI–PD subjects (*p* < 0.01). However, while the mean score of the UPDRS-motor scale (UPDRS-Part III) was higher in TBI+/PD vs. TBI–/PD subjects across all conditions, comparisons among conditions in the TBI+/PD group only did not reach a statistical significance (after multiple comparison adjustment) for differences between TBI+/PD/MCI and TBI+/PD/Impaired (*p* = 0.824), or between TBI+/PD/Dementia and TBI+/PD/Impaired (*p* = 0.072) (Figure 4).

## Discussion

In contrast to longitudinal studies on brain aging and dementia (often establishing a previous history of TBI as a criterion of exclusion) there have been, historically, much fewer prospective studies focusing on the possible cognitive and non-cognitive consequences induced by TBI events. However, it will take a few years, or even decades, before a consistent amount of detailed longitudinally-collected clinical, cognitive, behavioral, genetic, and neuropathological data from TBI subjects will be available. Consequently, we hypothesized that it would have been worthwhile to explore some possible general demographic, cognitive and genotype-associated aspects on TBI and cognitive decline by cross-sectional analyses using existing databases that contain already a sufficient amount of information on subjects prospectively assessed for cognitive and non-cognitive domains (e.g., motor, behavior), history of TBI, TBI time points, and APOE genotype frequencies (being the allele APOE4 is a well-known genetic risk factor for AD and other dementias) (39).

Among the most relevant findings of this investigation we found that a previous TBI exposure (which in our study included sTBI and rTBI, with and without LOC) at the time of the first cognitive decline assessment (TBI+ subjects), is associated with a decreased mean age of the onset of cognitive decline in comparison to subjects who did not report a history of TBI (TBI– subjects) at the same time point. This finding was not associated with any of the specific brain disorders considered (e.g., a specific type of dementia such as AD or DLB, or other specific neurodegenerative disorders such as PD), sex, race, attained educational level, or specific APOE genotype/allele frequency. Indeed, these data suggest that TBI exposure lowers the age, or alternatively, accelerates the manifestation of cognition decline irrespective of the incipient or already manifested subjacent neurodegenerative disease. In particular, these new findings show that the TBI-related age-lowering effect for cognitive decline is irrespective of the various molecular pathomechanisms predominantly involved in different disorders such as AD-pathology (1-42 β-amyloid neuritic plaques and hyperphosphorylated-tau-positive neurofibrillary-tau tangles) in amnesic-MCI and AD; phosphorylated-TDP43-inclusions and Lewy Body pathology, respectively, in FTD and DLB; presumably CTE or TBI-related pathologies in Impaired; or Lewy body pathology and pigmented neuronal loss in PD. Furthermore, the mean interval of time between the time of the first cognitive decline assessment and time of death was shortened in TBI+ vs. TBI– subjects across all considered clinical conditions and, again, independently of sex, age, education, and APOE genotype, suggesting a possible long-term and detrimental effect of TBI on survival or longevity in general (40).

These novel outcomes support the hypothesis that a previous history of TBI (of any type, and although the contributing “weight” of a specific type of TBI could not be completely excluded) is a clinically relevant *age-lowering factor* that determines an earlier manifestation of cognitive decline independently of the specific type of neurodegenerative disorder associated with or causing those cognitive abnormalities. A history of TBI seems to potentially represent then a more general detrimental factor for any subjacent or upcoming disorder (e.g., MCI, AD, FTD, DLB, PD), or alternatively, represent a specific trigger for those pathologies related to TBI events such as CTE, diffuse axonal injury (DAI), neuroinflammation, or a combination of them, generated at the time of a single or multiple TBI events, which could overlap with other subjacent or later incoming neurodegenerative processes.

The main limitations of our analyses are represented by: (a) the size of the examined subject cohorts, which did not allow the generalization of the concept that a previous TBI is indeed a global age-lowering factor for different, if not all, neurodegenerative disorders (including sporadic and genetic disorders); (b) the absence of sufficient amounts of data to better define the age-lowering effect of TBI based on clinical severity (e.g., including clinical staging, MRI analyses, biomarkers, and other non-APOE genetic data) and specific TBI types; (c) the unavailability of the exact age for each subject at the time of the first and following TBI exposure, and consequently the impossibility to measure time intervals between TBI and onset of the cognitive decline, which would represent another valuable information for different clinical, pathogenetic, and therapeutic aspects; (d) the source of the subjects analyzed, which were almost exclusively subjects enrolled in studies focusing on aging-related disorders analyses and not offering so the opportunity to generalize our findings to the general adult population.

By contrast, as points of strength, our investigation showed that TBI+ subjects did manifest cognitive decline earlier than TBI– subjects and that there were no associations with a specific neurodegenerative process, demographic factor (e.g., sex, race, education), or with a higher frequency of a specific APOE genotype or APOE allele. This latter finding, in particular, seems to exclude the hypothesis that the accelerator effect of TBI on the manifestation of cognitive decline might be due only or mainly, to the detrimental effect of APOE4, which remains, though, the major well-known genetic risk factor for AD, AD onset, AD clinical and pathological severity, and DLB (41–43).

As for some more specific cognitive outcomes observed in these TBI+ vs. TBI– NACC's cohorts, it appears that only very specific cognitive domains, such as those measured by the Part A of Trail Making test (frontal cortex functions) or the Boston naming test (language skills) can distinguish between subjects with cognitive decline due to TBI vs. cognitive decline due to other causes (e.g., MCI or AD). However, determining the specificity and sensitivity of the cognitive tests used in our analyses for the assessment of cognitive decline induced by TBI was outside the scope of this study. Yet, it should be important to emphasize that more specific TBI-oriented cognitive tests are indeed necessary to identify in order to assess distinct cognitive domains or sub-domains more frequently, particularly vulnerable, or especially predisposed to the TBI effects in contrast with cognitive domains or sub-domains more typically affected in MCI, AD, DLB, FTD, or other neurodegenerative conditions. Ideally, these cognitive TBI-oriented tests should be able to even separate, or identify at least, cognitive deficits of different types among different types of TBI [blunt-TBI vs. blast-TBI, or motor vehicle accident (MVA)-TBI vs. contact sports-TBI, for example] (44–47).

Also, it is of special interest to observe that PD/Impaired subjects with a previous history of TBI exposure (TBI+/PD/Impaired) appeared to have a higher frequency of a wide series of neuropsychiatric phenomena such as delusions, agitation/aggression, anxiety, apathy, disinhibition, nighttime behaviors, and motor disturbance in comparison to PD/Impaired subjects without a previous history of TBI at the time of the first cognitive decline assessment (TBI–/PD/Impaired). Specifically, the majority of these psychiatric symptoms are clinical phenomena usually linked to some of neuroanatomical regions more often affected by TBI events such as frontal, temporal, and cingulate cortex. Furthermore, these findings are in support of the recent data showing a significant correlation between history of TBI with LOC and Lewy body pathology accumulation (36).

Regrettably, the current NACC's TBI cohorts did not have sufficient amounts of neuropathological data to perform meaningful statistical analyses and identify, for example, specific clinico-pathological correlations between a peculiar type of TBI and AD pathology severity (e.g., MVA-TBI and hyperphosphorylated-Tau neurofibrillary tangles or β-amyloid neuritic plaques scores), or between TBI types and non-AD pathology loads or histological distribution (e.g., MVA-TBI vs. contact sports-TBI and Lewy bodies or TDP43 inclusions loads in specific traumatized regions of the brain). However, previous neuropathological studies appear to be consistent with our new findings (48–50). While TBI exposure appears to be a possible general detrimental factor for an anticipated manifestation of cognitive decline across different pathological conditions, we cannot either totally exclude that this TBI-associated risk factor could be determined, enhanced, or modulated by other factors (genetic, epigenetic, environmental, etc.), which could increase the risk for an earlier clinical manifestation of AD or non-AD conditions in the general population or in specific categories of predisposed subjects. Similarly, we cannot either exclude the possibility that some beneficial or positively factors (genetic, epigenetic, environmental, or other) could reduce, delay, or stop the manifestations of TBI-related cognitive decline or behavioral disorders in the contest of a specific disease (AD, for example), or across a group of disorders with a common pathogenetic basis (DLB and PD, for example).

Our findings are consistent with the hypothesis that TBI exposure is a risk factor not for a specific type of cognitive decline associated with a specific neurodegenerative disorder (e.g., AD), but rather is a risk factor for the anticipation of cognitive decline across different pathological neurodegenerative conditions (AD, FTD, DLB, PD, etc.). Alternatively, TBI could represent a risk factor for the initiation and progression of a specific TBI-induced pathology (e.g., CTE, traumatic axonal injury, neuroinflammation, etc.), or a combination of some degree of all of them, mixed with other incipient or already occurring brain pathologies.

Additionally, our data suggest that the “traditional” cognitive tests commonly used in the context of sporadic and genetic dementias or MCI as tools for the assessment of various cognitive domains (e.g., episodic memory, visuo-spatial skills, abstract thinking, language, etc.) do not efficiently discriminate between specific aspects of the cognitive deficits due to TBI vs. non-TBI processes (e.g., aMCI, AD, DLB, PD, and other), except for those cognitive domains or subdomains gauged by the TMT part A and Boston Naming Test.

Large longitudinal studies and dataset collections are needed to consolidate our initial observations. More detailed longitudinal clinico-imaging-pathological correlations between TBI+ vs. TBI– subjects as based on the specific features of their cognitive decline (specific affected cognitive domains, subdomains, operational skills, cognitive speed tasks, etc.) and prevalent brain pathologies, or co-pathologies, associated with (tauopathies, Lewy Body pathology, traumatic axonal injury, neuroinflammation, and other) are necessary. Indeed, the data available for this investigation did not allow to perform correlative analyses among different types of TBI (e.g., MVA vs. contact sports vs. falls), clinical and cognitive sequelae (spatio-temporal disorientation, memory loss, chronic headache, increased pain sensitivity, balance disorders, sleep disorders, etc.), autopsy-verified underlining brain pathologies, and specific types of cognitive and non-cognitive domains affected. For this reason, we would like to emphasize that future prospective TBI studies should include not only standard demographic data, but also detailed information about the time (the exact date) of the sTBI or rTBI occurrence/s, precise information about medical or health conditions before and at the time of the TBIs, data recordings from any TBI-validated clinico-metrics apparatus (including digital devices and telemedicine tools) for a more precise measurements of the TBI severity, clinical and prognostic grading, sequential post-TBI MRI (both structural and functional) and PET-scan evaluations, rapid and highly TBI-sensitive neuropsychological evaluations performed during a shorter (days/weeks) and longer (months/years) periods of time after each TBI event, neurophysiology measurements (quantitative electroencephalography, magnetoencephalography, evoked potentials), blood samplings for biomarkers and genetic analyses, an autopsy-brain donation programs (even, or especially, when the TBI event was not considered the immediate cause of death). This latter possibility would be of great clinical and research importance to perform post-mortem MRI acquisitions in order to identify precise and quantitative autopsy-verified imaging-pathological correlations analyses (51).

In summary, this investigation suggests that among the long-term pathogenetic effects of TBI there is an earlier cognitive decline across sexes, races, and educational levels, which is not uniquely or necessarily associated with a specific neurodegenerative disorder, but rather represents a possible unfavorable global consequence that may be associated with manifestations of cognitive decline and multiple behavioral abnormalities in the context of AD and non-AD conditions. Alternatively, TBI may be only an additional detrimental factor for other incipient or clinically manifested neurodegenerative processes, not primarily induced by and pathophysiologically unrelated to a previous history of TBI (52).

## Data Availability Statement

The raw data supporting the conclusions of this article will be made available by the authors, without undue reservation.

## Author Contributions

DI and SR contributed to the conception, design of the study, drafting the text, and preparing the figures. DP and CO contributed to the data and analyses review. All authors contributed to the acquisition and analyses of data.

## Conflict of Interest

The authors declare that the research was conducted in the absence of any commercial or financial relationships that could be construed as a potential conflict of interest.
