# Supplementary material for: Traumatic Brain Injury Exposure Lowers Age of Cognitive Decline in AD and Non-AD Conditions
Source: Front Neurol. 2021 May 12;12:573401. doi: 10.3389/fneur.2021.573401 (PMC8153372; doi:10.3389/fneur.2021.573401)
Supplement: Supplementary file 1 [file Data_Sheet_1.DOCX]

| **Supplementary Data** |
| --- |
| **Demographics Variables**  NACCADC NACCID FORMVER PACKET NACCVNUM VISITNUM VISITMO VISITDAY VISITYR SEX NACCAGE NACCNIHR EDUC  **TBI Variables**  TBI TBIBRIEF TRAUMBRF TBIEXTEN TRAUMEXT TBIWOLOS TRAUMCHR TBIYEAR BRNINJ BRNINJIF  **Neurological Examination Variables**  ABRUPT STEPWISE SOMATIC EMOT HXHYPER HXSTROKE FOCLSYM FOCLSIGN HACHIN CVDCOG STROKCOG CVDIMAG CVDIMAG1 CVDIMAG2 CVDIMAG3 CVDIMAG4 CVDIMAGX PDNORMAL SPEECH SPEECHX FACEXP FACEXPX TRESTFAC TRESTFAX TRESTRHD TRESTRHX TRESTLHD TRESTLHX TRESTRFT TRESTRFX TRESTLFT TRESTLFX TRACTRHD TRACTRHX TRACTLHD TRACTLHX  RIGDNECK RIGDNEX RIGDUPRT RIGDUPRX RIGDUPLF RIGDUPLX RIGDLORT RIGDLORX RIGDLOLF RIGDLOLX TAPSRT TAPSRTX  TAPSLF TAPSLFX HANDMOVR HANDMVRX HANDMOVL HANDMVLX HANDALTR HANDATRX HANDALTL HANDATLX LEGRT LEGRTX  LEGLF LEGLFX ARISING ARISINGX POSTURE POSTUREX  GAIT GAITX POSSTAB POSSTABX BRADYKIN BRADYKIX MEMORY ORIENT JUDGMENT COMMUN HOMEHOBB PERSCARE CDRSUM C DRGLOB C OMPORT CDRLANG FOCLDEF GAITDIS  EYEMOVE PARKSIGN RESTTRL RESTTRR SLOWINGL SLOWINGR RIGIDL RIGIDR BRADY PARKGAIT POSTINST CVDSIGNS  CORTDEF SIVDFIND CVDMOTL CVDMOTR CORTVISL CORTVISR  SOMATL SOMATR POSTCORT PSPCBS EYEPSP DYSPSP  AXIALPSP GAITPSP APRAXSP APRAXL APRAXR CORTSENL CORTSENR ATAXL ATAXR ALIENLML ALIENLMR DYSTONL DYSTONR MYOCLLT MYOCLRT ALSFIND GAITNPH OTHNEUR OTHNEURX  **Cognition, Neuropsychiatry and Imaging Variables**  MMSECOMP MMSELOC MMSELAN MMSELANX MMSEVIS MMSEHEAR MMSEORDA MMSEORLO PENTAGON NACCMMSE NPSYCLOC NPSYLAN NPSYLANX LOGIMO LOGIDAY LOGIYR LOGIPREV LOGIMEM MEMUNITS MEMTIME UDSBENTC UDSBENTD UDSBENRS DIGIF DIGIFLEN DIGIB DIGIBLEN ANIMALS VEG TRAILA TRAILARR TRAILALI TRAILB TRAILBRR TRAILBLI WAIS  BOSTON UDSVERFC UDSVERFN UDSVERNF UDSVERLC UDSVERLR UDSVERLN UDSVERTN UDSVERTE UDSVERTI COGSTAT NACCC1  MOCACOMP MOCAREAS MOCALOC MOCALAN MOCALANX MOCAVIS MOCAHEAR MOCATOTS MOCATRAI MOCACUBE MOCACLOC MOCACLON MOCACLOH MOCANAMI MOCAREGI MOCADIGI MOCALETT MOCASER7 MOCAREPE MOCAFLUE MOCAABST MOCARECN MOCARECC MOCARECR MOCAORDT MOCAORMO MOCAORYR MOCAORDY MOCAORPL MOCAORCT CRAFTVRS CRAFTURS DIGFORCT DIGFORSL DIGBACCT DIGBACLS CRAFTDVR CRAFTDRE CRAFTDTI CRAFTCUE MINTTOTS MINTTOTW MINTSCNG MINTSCNC MINTPCNG MINTPCNC NACCC2 WHODIDDX DXMETHOD NORMCOG DEMENTED AMNDEM PCA NACCPPA NACCPPAG NACCPPME NACCBVFT NACCLBDS NAMNDEM NACCTMCI NACCMCIL NACCMCIA NACCMCIE NACCMCIV NACCMCII IMPNOMCI AMYLPET AMYLCSF FDGAD HIPPATR TAUPETAD CSFTAU  FDGFTLD TPETFTLD MRFTLD DATSCAN OTHBIOM OTHBIOMX IMAGLINF IMAGLAC IMAGMACH IMAGMICH IMAGMWMH IMAGEWMH OTHMUT OTHMUTX  NPIQINF NPIQINFX DEL DELSEV HALL HALLSEV AGIT AGITSEV DEPD DEPDSEV ANX ANXSEV  ELAT ELATSEV APA APASEV DISN DISNSEV  IRR IRRSEV MOT MOTSEV NITE NITESEV  APP APPSEV NOGDS SATIS DROPACT EMPTY  BORED SPIRITS AFRAID HAPPY HELPLESS STAYHOME MEMPROB WONDRFUL WRTHLESS ENERGY HOPELESS BETTER NACCGDS BILLS TAXES SHOPPING GAMES STOVE MEALPREP EVENTS PAYATTN REMDATES TRAVEL NACCNREX NORMEXAM  **Neurological Diagnosis Variables**  NACCALZD NACCALZP PROBAD PROBADIF POSSAD POSSADIF NACCLBDE NACCLBDP PARK MSA MSAIF PSP PSPIF CORT CORTIF FTLDMO FTLDMOIF FTLDNOS FTLDNOIF FTD FTDIF PPAPH PPAPHIF FTLDSUBT FTLDSUBX CVD CVDIF PREVSTK STROKDEC STKIMAG INFNETW INFWMH VASC VASCIF VASCPS VASCPSIF STROKE STROKIF ESSTREM ESSTREIF DOWNS DOWNSIF HUNT HUNTIF PRION PRIONIF BRNINCTE HYCEPH HYCEPHIF EPILEP EPILEPIF NEOP NEOPIF NEOPSTAT HIV HIVIF OTHCOG OTHCOGIF OTHCOGX DEP DEPIF DEPTREAT BIPOLDX BIPOLDIF SCHIZOP SCHIZOIF ANXIET ANXIETIF DELIR DELIRIF PTSDDX PTSDDXIF OTHPSY OTHPSYIF OTHPSYX ALCDEM ALCDEMIF ALCABUSE IMPSUB IMPSUBIF DYSILL DYSILLIF MEDS MEDSIF DEMUN DEMUNIF COGOTH COGOTHIF COGOTHX COGOTH2 COGOTH2F COGOTH2X COGOTH3 COGOTH3F COGOTH3X NACCETPR NACCADMU NACCFTDM NACCNORM NACCIDEM NACCUDSD NACCNE4S NACCAPOE NACCAUTP NACCFTD NACCBNKF NACCFORM NACCPARA |
